# Harnessing the frontal aslant tract’s structure to assess its involvement in cognitive functions: new insights from 7-T diffusion imaging

**DOI:** 10.1038/s41598-024-67013-w

**Published:** 2024-07-29

**Authors:** Lucas Serrano-Sponton, Felipa Lange, Alice Dauth, Harald Krenzlin, Ana Perez, Elke Januschek, Sven Schumann, Daniel Jussen, Marcus Czabanka, Florian Ringel, Naureen Keric, Gabriel Gonzalez-Escamilla

**Affiliations:** 1 Department of Neurosurgery, Sana Clinic Offenbach, Johann Wolfgang Goethe University Frankfurt am Main Academic Hospitals, Starkenburgring 66, 63069 Offenbach am Main, Germany; 2 Department of Neurosurgery, University Medical Center of the Johannes Gutenberg University Mainz, Langenbeck Str. 1, 55131 Mainz, Germany; 3 Department of Neurology, Oslo University Hospital HF, Sognsvannsveien 20, 0372 Oslo, Norway; 4 Institute of Anatomy, University Medical Center of the Johannes Gutenberg-University Mainz, Johann-Joachim-Becher-Weg 13, 55128 Mainz, Germany; 5 Department of Neurosurgery, University Medical Center of the Johann Wolfgang Goethe University Frankfurt am Main, Theodor-Stern-Kai 7, 60590 Frankfurt am Main, Germany; 6 Movement Disorders and Neurostimulation, Department of Neurology, Focus Program Translational Neuroscience, Rhine Main Neuroscience Network, University Medical Center of the Johannes Gutenberg University Mainz, Langenbeck Str. 1, 55131 Mainz, Germany

**Keywords:** Frontal aslant tract, Episodic memory, Visuospatial orientation, Cognitive processing speed, Fluid intelligence, Sustained attention, Neuroscience, Anatomy, Neurology

## Abstract

The first therapeutical goal followed by neurooncological surgeons dealing with prefrontal gliomas is attempting supramarginal tumor resection preserving relevant neurological function. Therefore, advanced knowledge of the frontal aslant tract (FAT) functional neuroanatomy in high-order cognitive domains beyond language and speech processing would help refine neurosurgeries, predicting possible relevant cognitive adverse events and maximizing the surgical efficacy. To this aim we performed the recently developed correlational tractography analyses to evaluate the possible relationship between FAT’s microstructural properties and cognitive functions in 27 healthy subjects having ultra-high-field (7-Tesla) diffusion MRI. We independently assessed FAT segments innervating the dorsolateral prefrontal cortices (dlPFC-FAT) and the supplementary motor area (SMA-FAT). FAT microstructural robustness, measured by the tract’s quantitative anisotropy (QA), was associated with a better performance in episodic memory, visuospatial orientation, cognitive processing speed and fluid intelligence but not sustained selective attention tests. Overall, the percentual tract volume showing an association between QA-index and improved cognitive scores (pQACV) was higher in the SMA-FAT compared to the dlPFC-FAT segment. This effect was right-lateralized for verbal episodic memory and fluid intelligence and bilateralized for visuospatial orientation and cognitive processing speed. Our results provide novel evidence for a functional specialization of the FAT beyond the known in language and speech processing, particularly its involvement in several higher-order cognitive domains. In light of these findings, further research should be encouraged to focus on neurocognitive deficits and their impact on patient outcomes after FAT damage, especially in the context of glioma surgery.

## Introduction

The frontal aslant tract (FAT) is a white matter bundle connecting the supplementary motor area (SMA), as well as portions of the dorsolateral prefrontal cortex (dlPFC), with the inferior frontal gyrus, the frontal operculum, anterior insula and the inferior portion of the precentral gyrus^1–7^. Since its relatively recent descriptions about a decade ago^7,8^, neuropsychological and imaging studies, as well as clinical observations, especially after the resection of brain tumors infiltrating the tract, have documented a clear involvement of the left FAT in speech and language^1,9–14^. The left FAT has been observed to play a role in semantic and phonemic fluency, speech programming, controlled lexical and phonological selection/retrieval, syntactic processing and fluent speech^1,15^. On the contrary, although there is also some evidence regarding the participation of the right FAT in working memory and visuomotor executive functions^1,15,16^, the involvement of FAT in other higher order cognitive functions has remained unattended in the published literature and still remains to be fully elucidated. This knowledge constitutes a relevant issue in neurosurgery, particularly in the context of glioma surgery, where the concept of supramarginal tumor resection should always be tailored against possible neurocognitive deficits with relevant consequences for the patient’s social and professional life. Therefore, research seeking further characterization of the putative role played by the FAT in cognition is needed.

First, the possible relationship between the FAT and mnemonic processes has yet to be thoroughly explored. The frontal lobe’s integrity is essential for working memory (WM) performance^17–19^, a function described as the capability to retain and manipulate information temporarily to ensure successful goal-directed task execution^20^. Being the FAT a robust frontal intralobar associative pathway, it is not surprising that its involvement in WM performance has been documented^6,21–23^. However, there is also consistent evidence demonstrating the involvement of the frontal lobe, not only in WM processes, but also in other mnemonic functions, such as in the case of episodic memory (EM), known as the capability to store and recall specific events that might be related to verbal or non-verbal (primarily visual) information^24^. Although the temporal lobe is well known to play the central role in EM acquisition^24–28^, the frontal lobe has been linked to mechanisms of EM recall and top-down modulation during EM storage^7,29–32^. Supporting this evidence, patients with frontal lobe tumors tend to present EM deficits with a detrimental impact on their quality of life^33,34^. The possible role played by the FAT in these (less studied) frontal lobe mechanisms of EM processing, which could partly explain the EM deficits shown by these patients, has not been established yet.

In addition, early studies have already pinpointed a possible involvement of the right FAT in executive functions, including visuospatial-motor integration between limb movements and the oculomotor systems, including inhibitory control^15,16,35,36^. But, whether the FAT may be involved in earlier mechanisms subserving visuospatial-motor integration, such as spatial orientation and sustained visual attention, remains unclear. Similarly, even when the FAT has been linked to executive functions, working memory and fluent speech, the possible involvement of the FAT in closely related functions such as cognitive processing speed (defined as a more general domain related to the speed with which information is mentally processed)^37^ or intelligence has been scarcely studied yet.

In order to evaluate the role of FAT on these cognitive functions, we used ultra-high-field diffusion 7 T-MRI and cognitive data from the human connectome project (HCP) to assess an eventual implication of the FAT robustness in verbal and non-verbal EM, visuospatial orientation, sustained visual attention, cognitive processing speed and fluid intelligence. The results may contribute to further understanding of the putative role played by the FAT in cognition, helping to delineate future research considering these aspects for cognitive testing in the context of brain tumor surgery.

## Methods

### Creation of a connectometry database

We created a connectometry database using preprocessed 7 T diffusion MRI data of HCP participants, available from https://db.humanconnectome.org (assessed on 01/31/2023). The Human Connectome Project, WU-Minn Consortium consists in a large open access MRI database, which has been created by the 16 NIH Institutes and Centers that support the NIH Blueprint for Neuroscience Research and by the McDonnell Center for Systems Neuroscience at Washington University, fulfilling all ethical standards according to the declaration of Helsinki. For HCP datasets, ethical approval has been given by the Washington University Institutional Review Board and patients/participants provided their written inform consent to participate in the project. We agreed to Data Use Terms for the HCP before being allowed access to the data and ConnectomeDB; details are provided at https://www.humanconnectome.org/study/hcp-young-adult/data-use-terms. MRI and cognitive data used were anonymized, so that there is no risk of identification. Methods were carried out in accordance with current guidelines, regulated in the Rhineland-Palatinate State by the Hospital Law §36, §37 and no ethical concerns for this study were observed by the Ethics Committee of the Johannes Gutenberg University Mainz.

The HCP MRI data pre-processing pipeline consists of a combination of FSL and FreeSurfer and described elsewhere^38–41^. Diffusion imaging distortion correction techniques applied to all HCP open-access pre-processed diffusion data are also described elsewhere^42–44^. A complete anonymized behavioral and cognitive dataset was available for all screened subjects. In order to avoid possible bias arising from declined baseline cognition or depressive behavior that were not accountable, could not be counterbalanced, therefore rendering our analyses less robust, the following criteria were applied for subject selection: mini-mental-status-examination (MMSE) scores > 28, NIH toolbox sadness survey < 50, NIH toolbox general life satisfaction survey > 50, NIH toolbox meaning and purpose survey > 50, NIH toolbox positive affect survey > 50; and NIH toolbox loneliness survey < 50 (for score interpretation see the Supplementary information). After screening, a cohort of 27 healthy subjects (not biased by inhomogeneous emotional or baseline cognitive state) was included in the study (female N = 16, male N = 11; all subjects aged between 22 and 35 years old).

### Cognitive tests

The following cognitive scores, from computer-based tests, were collected for each subject from the HCP database.

#### Penn word memory test (PWMT)^45,46^

The PWMT is a measure of episodic memory for verbal material. Participants are shown 20 words that they will be asked to identify later at 20 min delay. During the recognition phase, participants are shown a series of 40 words, 20 of which are the stimuli they were asked to memorize, and the other 20 are distractors. The participant's score reflects the number of correctly recognized targets and correctly rejected foils. Median response times for correct responses serve as a measure of processing speed. The total number of correct responses (TOT) and median reaction time for correct responses (RTC) are recorded.

#### Variable short Penn line orientation test (VSPLOT)^45,46^

The VSPLOT is a valid measure for visuospatial processing with minimal motor or language demands. It has been considered a novel method that combines item response theory (IRT) and computerized adaptive testing (CAT) approaches^47^. There are a total of 15 trials in the test. Two-line segments are presented on the screen and participants are asked to rotate a movable line so that it is parallel to the fixed line. The number of degrees of rotation for each click varies producing increased precision demand, and thereby increasing the level of difficulty. In each trial, the location of the lines relative to one another varies, but the distance between the centers remains constant. The length of the movable line also varies among three lengths in different trials, but the length of the fixed line remains constant. The total number of correct trials (TC) and the median reaction time divided by the expected number of clicks for correct (CRTE) are recorded. Furthermore, the total clicks of orientation positions “off” from the correct one (correct considered parallel to the reference line) the participant has across all 24 trials are recorded as the total positions “off” for all trials (OFF). Thus, VSPLOT_OFF asserts how inefficient/incorrect the orientation matching was for that participant over all trials.

#### Short Penn continuous performance test (SPCPT)^45,46^

The SPCPT is a brief computerized test that measures visual sustained attention and vigilance independent of working memory or perceptual factors. It consists of two blocks of trials, each lasting 1.5 min. In each block, participants are presented with a series of rapid-fire stimuli (vertical and horizontal lines) on a computer screen. Participants are instructed to press a button whenever they see a specific target stimulus (e.g., a number or a letter) and have 1 s to respond to each trial. The SPCPT is scored based on the number of correct responses and the number of errors of omission (targets that were missed) and commission (non-targets that were incorrectly identified as targets).

#### Penn progressive matrices reasoning test (PMRT)^45,46^

The PMRT measures fluid intelligence via non-verbal reasoning using an abbreviated version of Raven's progressive matrices form A^48^. Participants are presented with patterns of 2 × 2, 3 × 3 or 1 × 5 arrangements of squares, with one of the squares missing. The participant must pick one of five response choices that best fit the missing square on the pattern. The task has 24 items and three bonus items, arranged in order of increasing difficulty. However, the task discontinues if the participant makes five incorrect responses in a row. The number of correct responses (CR), total skipped items (SI) and median reaction time for correct responses (RTCR) are recorded.

#### The age-adjusted NIH toolbox picture sequence memory test (PSMT)^49^

This test is a measure for non-verbal episodic memory that has been validated for ages between 3 and 85 years. A series of images and activities are shown in a predetermined sequence while verbally describing them. Participants are required to memorize and recall the sequence over three learning trials. Sequence length varies from 6 to 18 pictures, depending on age. In the age-adjusted scale score, the individual participant score is normed using the age-appropriate band of Toolbox Norming Sample, where a score of 100 indicates performance that was at the national average and a score of 115 or 85, indicates performance 1 SD above or below the national average for participants age band.

#### Age-adjusted NIH toolbox pattern comparison cognitive processing speed test (Proc Speed)^49^

This test is an assessment of processing speed across lifespan. Participants are asked to quickly determine whether two side- by- side pictures presented on a computer screen, are the same or not. They are given 90 s to respond to as many items as possible (of a possible 130). The items are designed to be simple to measure processing speed. Patterns are either identical or varied on one of three dimensions: color, adding/taking something away, or one versus many. In the age-adjusted scale score, the participant score is normed using the age-appropriate band of the toolbox norming sample, where a score of 100 indicates performance that was at the national average and a score of 115 or 85 indicates performance 1 SD above or below the national average for participants age band.

### MRI acquisition and processing

A multishell diffusion scheme was used with b-values of 1000 and 1995 s/mm^2^. The number of diffusion sampling directions was 64 and 64, respectively. The in-plane resolution was 1.05 mm. The slice thickness was 1.05 mm. The accuracy of b-table orientation was examined by comparing fiber orientations with those of a population-averaged template^50^. The restricted diffusion was quantified using restricted diffusion imaging^51^. The diffusion data were reconstructed using generalized q-sampling imaging with a diffusion sampling length ratio of 1.25^51^. The tensor metrics were calculated using DWI with a b-value lower than 1750 s/mm^2^.

### FAT delineation and reconstruction

**Figure 1:**
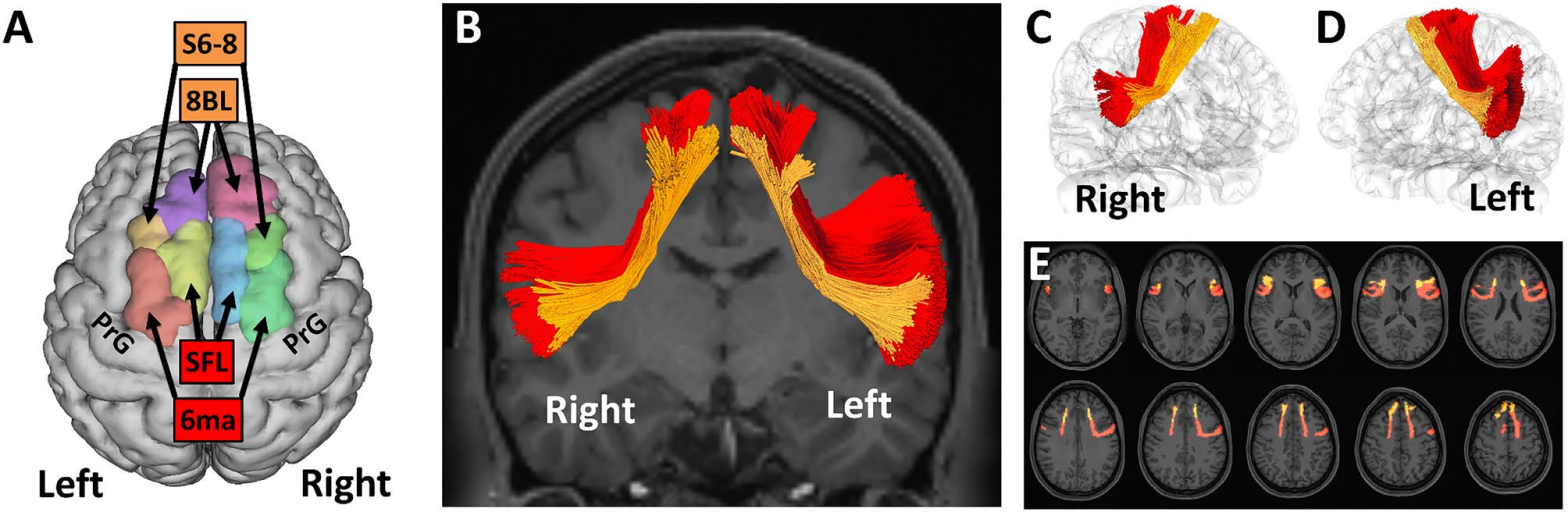
Frontal aslant tract (FAT) segmentation. Based on cortical HCP atlas, an automatic algorithm was used to identify the S6–8 and 8BL cortices (belonging to the dlPFC, orange), as well as SFL and 6ma cortices (belonging to the SMA, red), immediately anterior to the precentral gyrus (PrG) bilaterally (**A**). We applied a state-of-the-art automated deterministic tractography pipeline, to identify the FAT as a fiber bundle connecting the S6–8, 8BL, SFL and 6ma cortices with the IFG (parcellations 44, 6r), the frontal operculum (parcellations FOP1, FOP3, and FOP4), the middle insula (MI) parcellation in the anterior insula. A further differential FAT segmentation was done according to whether those fibers innervated either the dorsolateral prefrontal cortices S6–8 and 8BL (dlPFC-FAT, orange) or the supplementary motor area SFL and 6ma (SMA-FAT, red). Image (**B**) shows a bilateral coronal view of the SMA-FAT and dlPFC-FAT; images (**C**, **D**) show respectively the right and left FAT components in a brain’s isosurface reconstruction; and image E shows the SMA-FAT and dlPFC-FAT in axial slices.

According to the most recent annotations, the FAT was defined by fibers connecting the 6ma and SFL cortices (two parcellations of the SMA complex), as well as 8BL and S6–8 (belonging to the dlPFC^3,52^), with the IFG (parcellations 44, 6r), the frontal operculum (parcellations FOP1, FOP3, and FOP4), the middle insula (MI) parcellation in the anterior insula and the inferior region of the precentral gyrus (PrCG) (Fig. 1)^1–7^.

We firstly recorded the total FAT volume in both hemispheres. Given the complex FAT connectivity, to delve deeper into putative differences between FAT components and increase the detailedness of our analysis, we further segmented the FAT into a more anterior and posterior component to be analysed separately. In the lack of homogeneity regarding well established FAT segments, we decided to analyse separately the tract in those portions linked to the more posterior SMA cortices and the more anterior cortices belonging to the dlPFC. The rationale to subdivide the tract into these two antero-posterior segments was (1) the fact that although the ventral portions of the tract diversify into several cortices, the dorsal portions of the tract are clearly delimited to the S6–8 and 8BL cortices (belonging to the dlPFC) and SFL and 6ma cortices (belonging to the SMA); (1) and (2) the SMA and dlPFC constitute two well established subdivisions of the frontal neocortex with different patterns of connectivity network^53–56^. Therefore, we considered consistent to further analyse the tract according to the FAT component innervating the dorsolateral prefrontal cortices 8BL + S6–8 (dlPFC-FAT) and the one innervating the SMA-complex cortices 6ma + SFL (SMA-FAT) (Fig. 1). DSI-Studio was used for cortical and white matter segmentation and volumetric tract analysis. To accurately map the FAT bundles, we employed a state-of-the-art automated tractography pipeline, based on population-based tract-to-region connectome of the human brain and its hierarchical topology^57^. A deterministic fiber tracking algorithm was used with augmented tracking strategies to improve reproducibility^58,59^. The anisotropy threshold was randomly selected. The angular threshold was randomly selected from 15 to 90 degrees. The step size was randomly selected from 0.5 voxels to 1.5 voxels. Tracks with lengths shorter than 30 or longer than 200 mm were discarded. A total of 1,000,000 seeds were placed. Topology-informed pruning was applied to the tractography with two iterations to remove false connections^60^. A final manual semiautomatic refinement was applied to remove fibers belonging to other overlapping tracts.

### Connectometry analysis

Based on robust pervious evidence showing that surrogate parameters for the white matter tract’s microstructural robustness correlate and underlie cognitive performance in a broad spectrum of MRI studies^61–65^, we used the already validated methods of diffusion MRI connectometry to derive the correlational tractography that has quantitative anisotropy with each of the above-mentioned cognitive scores^66^. QA was used as a measure of axonal density and white matter microstructural robustness and was extracted as the local connectome fingerprint^67^. The nonparametric Spearman method was used to derive the correlation. A total of 27 subjects were included in the analysis. A T-score threshold of 2.5 was assigned and tracked using a deterministic fiber tracking algorithm to obtain correlational tractography^59^. An ROI was placed separately at the FAT right and left based on the HCP tractography atlas. A seeding region was placed at the whole brain. The tracks were filtered by topology-informed pruning with 16 iterations^60^. A false discovery rate (FDR) threshold of 0.05 was used to select tracks. To estimate the FDR, 4000 randomized permutations were applied to the group label to obtain the null distribution of the track length.

The volume of FAT fibers showing association (either positive or negative), with each cognitive score was recorded as QA-cognitive-correlating volume (QACV). Given FAT asymmetric volume between right and left, as well as between anterior (dlPFC-FAT) and posterior (SMA-FAT) segments, we calculated the percentage of the tract (and tract-segment) volume represented by the QACV (pQACV), by dividing the QACVs by their corresponding cohort’s mean FAT volume, multiplied by 100. The same principle was applied separately for the SMA-FAT and dlPFC-FAT components (i.e., QACV of the SMA-FAT/cohort’s mean SMA-FAT volume × 100), as well as right-left FAT (i.e., QACV of the right SMA-FAT/cohort’s mean right SMA-FAT volume × 100). The obtention of pQACV was necessary to compare the QACV corresponding to the FAT and its dlPFC and SMA segments between right and left, as well as the dlPFC-FAT and SMA-FAT within the same hemisphere, avoiding bias arising from differential and asymmetrical FAT (and FAT segment) volumes.

### Statistical analysis

DSI Studio (Version 12/22/2022) was used for correlational tractography analysis, GraphPad Prism Version 9.4.0 (GraphPad Software, Inc., San Diego, CA) was used for further statistical analysis. Unpaired T-tests were used for assessing FAT-volume differences between hemispheres and FAT components (*p < 0.05; **p < 0.01; ***p < 0.001; ****p < 0.0001). Contingency tables and two-tailed chi-squared tests were applied for statistical comparisons between pQACVs. In cases of frequencies lower than 5%, Yates correction was applied. After correcting for multiple comparisons, an adjusted alpha level of 0.00076 was settled, and the null hypothesis was rejected only for p-values lower than this (*p < 0.00076).

## Results

**Figure 2:**
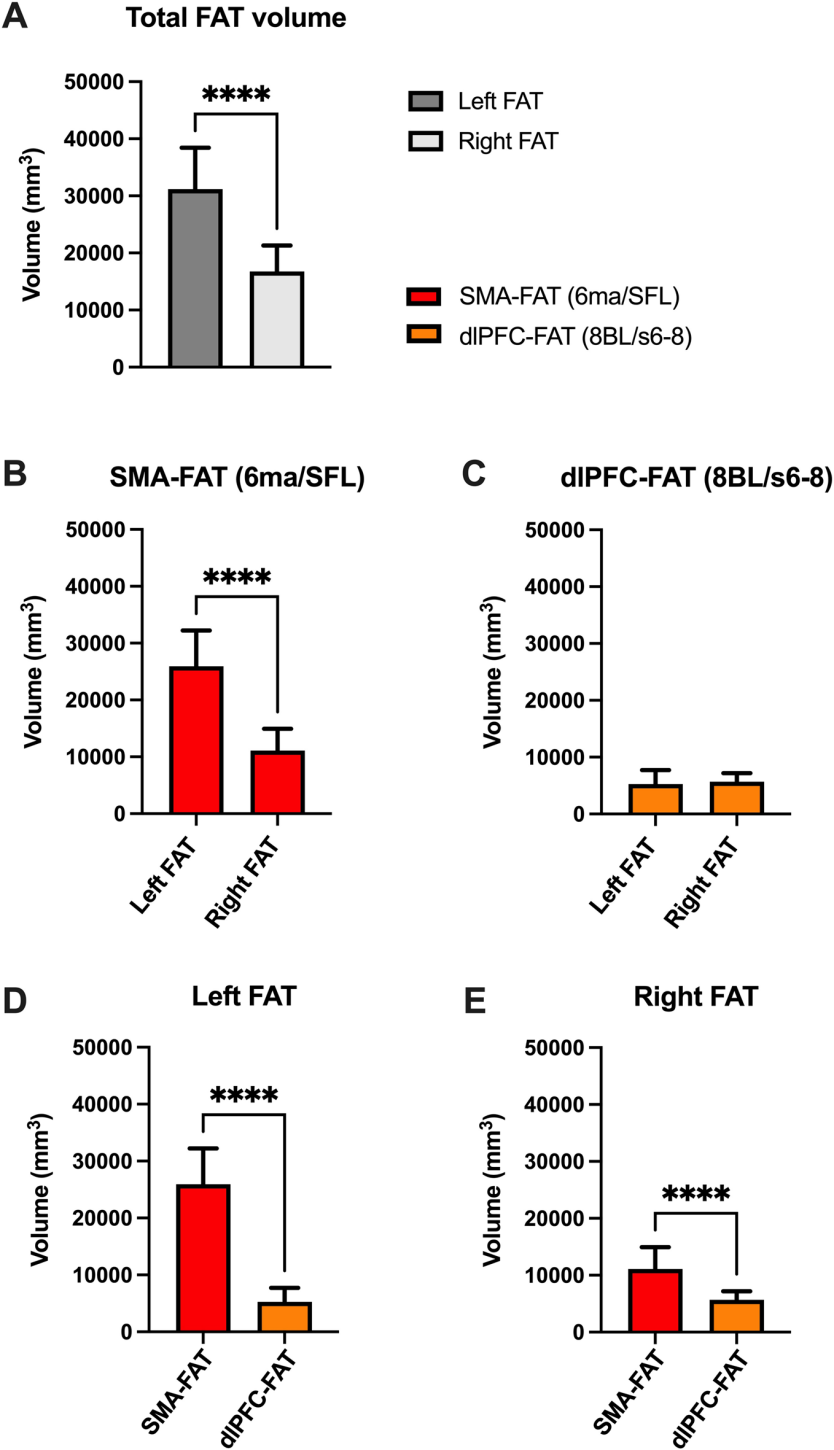
Frontal aslant tract (FAT) volumetry. Unpaired T-tests were employed to assess volumetric differences between the left and right FAT volume (**A**), as well separately for the SMA-FAT and dlPFC-FAT components (**B**, **C**, respectively). Further unpaired T-tests revealed the volumetric differences between the SMA-FAT and dlPFC-FAT within the same hemisphere (**D**, **E**). Bars represent means ± 2 standard error.

FAT volume was significantly more prominent in the left hemisphere compared to the right one (p < 0.0001, Fig. 2A). This difference was attributable to the significantly larger left SMA-FAT volume than its analogous SMA-FAT on the right side (p < 0.0001, Fig. 2B). The volumes corresponding to the dlPFC-FAT were similar in both hemispheres, with no statistically significant differences between them (p = 0.4481, Fig. 2C). SMA-FAT volume was significantly larger in both hemispheres than the dlPFC-FAT component (p < 0.0001, Fig. 2D,E).

**Figure 3:**
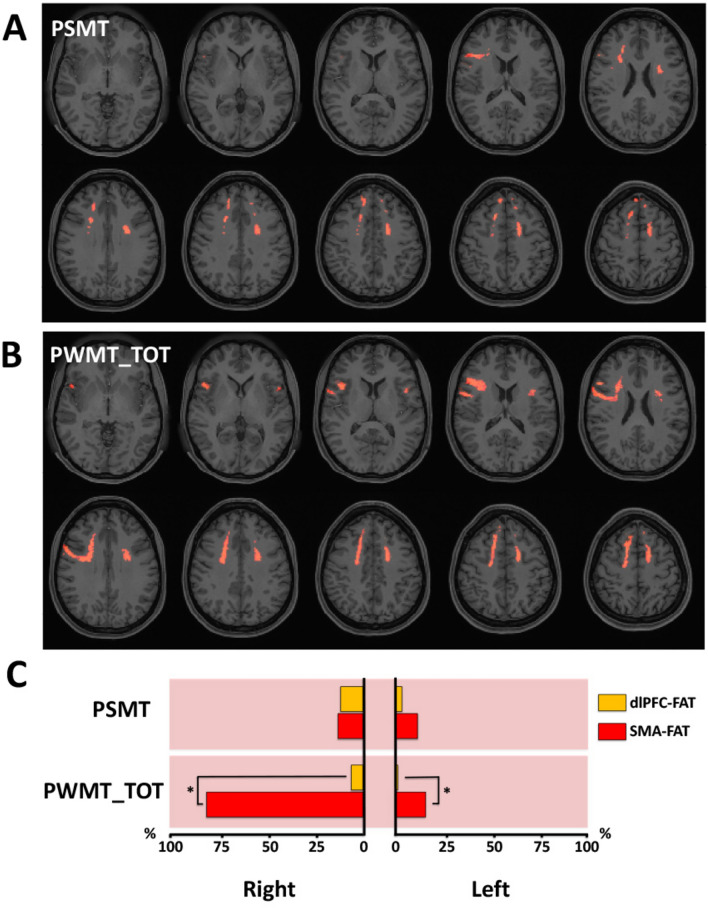
Correlational tractography between FAT-QA-index and episodic memory (EM) scores. Employing diffusion MRI connectometry^56^, a correlation between the QA-index in FAT fibers and non-verbal and verbal EM scores was derived for the total of 27 subjects. Axial slices in (**A**, **B**) illustrate FAT fibers showing association between increased QA and higher scores in the age-adjusted NIH toolbox picture sequence memory test (PSMT), and total number of correct responses in the Penn word memory test (PWMT_TOT), respectively (red indicating positive association). As depicted in (**C**), we calculated the percentual volume of the dlPFC-FAT (orange bars) and SMA-FAT (red bars) showing QA-PiqSeq-score and QA-PWMT_TOT-score association (pQACV). Pink-shadowed charts indicate positive association. Chi-squared tests were applied for statistical comparisons of pQACV between both FAT subsegments.

An association between increased QA and higher scores in non-verbal episodic memory test was found involving 11% of the total FAT volume (Fig. 3A,C). There was no statistically significant difference regarding pQACV between hemispheres. All pQACVs are depicted in Table 1.

**Table 1:** Percentual volumes showing association between increased QA-index and the scores obtained in each cognitive metric (pQACV).

Cognitive test	FAT (pQACV)				SMA-FAT (pQACV)				dlPFC-FAT (pQACV)				Left and right FAT (pQACV)				Left FAT (pQACV)				Right FAT (pQACV)			
	Left	Right	Chi-square	*p*	Left	Right	Chi-square	*p*	Left	Right	Chi-square	*p*	SMA-FAT	dlPFC-FAT	Chi-square	*p*	SMA-FAT	dlPFC-FAT	Chi-square	*p*	SMA-FAT	dlPFC-FAT	Chi-square	*p*
**PSMT**	**9**	**13**	**0.817**	**0.3660**	**11**	**13**	**0.1890**	**0.6634**	**3**	**12**	**4.6130**	**0.0317**	**11**	**8**	**0.5230**	**0.4694**	**11**	**3**	**3.7630**	**0.0524**	**13**	**12**	**0.0460**	**0.8307**
**PWMT _TOT**	**13**	**55**	**39.305**	< 0.0001*	**15**	**81**	**87.2600**	< 0.0001*	**1**	**6**	**2.3690**	**0.1238**	**35**	**3**	**31.2220**	< 0.0001*	**15**	**1**	**11.4810**	0.0007*	**81**	**6**	**114.4340**	< 0.0001*
**PWMT _RCT**	**9**	**3**	**2.216**	**0.1366**	**10**	**1**	**6.1570**	**0.0131**	**0**	**9**	**7.4460**	**0.0064**	**7**	**5**	**0.3550**	**0.5515**	**10**	**0**	**8.5260**	**0.0035**	**1**	**9**	**5.1580**	**0.0231**
**VSPLOT_TC**	**17**	**31**	**5.373**	**0.0205**	**21**	**37**	**6.2170**	**0.0127**	**0**	**19**	**18.8430**	< 0.0001*	**26**	**10**	**8.6720**	**0.0032**	**21**	**0**	**21.2820**	< 0.0001*	**37**	**19**	**8.0360**	**0.0046**
**VSPLOT_CRTE**	**11**	**0**	**9.620**	**0.0019**	**13**	**0**	**11.8470**	*0.0006	**0**	**0**	**–**	**–**	**9**	**0**	**7.4460**	**0.0064**	**13**	**0**	**11.8470**	*0.0006	**0**	**0**	**–**	**–**
*VSPLOT_OFF*	*50*	*52*	*0.080*	*0.7773*	*56*	*73*	*6.3110*	*0.0120*	*16*	*10*	*1.5920*	*0.2071*	*62*	*13*	*51.2210*	< 0.0001*	*56*	*16*	*34.7220*	< 0.0001*	*73*	*10*	*81.7420*	< 0.0001*
**Proc Speed**	**26**	**20**	**1.016**	**0.3134**	**31**	**30**	**0.0240**	**0.8779**	**0**	**1**	**1.0050**	**0.3161**	**30**	**0**	**32.9800**	< 0.0001*	**31**	**0**	**34.3580**	< 0.0001*	**30**	**1**	**29.9290**	< 0.0001*
**PMRT_CR**	**12**	**47**	**29.451**	< 0.0001*	**13**	**57**	**42.5490**	< 0.0001*	**2**	**27**	**23.2300**	< 0.0001*	**27**	**15**	**4.3400**	**0.0372**	**13**	**2**	**7.2070**	**0.0073**	**57**	**27**	**18.4730**	< 0.0001*
*PMRT_SI*	*9*	*23*	*7.292*	*0.0069*	*10*	*34*	*16.7830*	< 0.0001*	*2*	*3*	*0.2050*	*0.6506*	*18*	*2*	*12.5000*	0.0004*	*10*	*2*	*4.3440*	*0.0371*	*34*	*3*	*29.8460*	< 0.0001*
**PMRT_RTCR**	**17**	**64**	**45.835**	< 0.0001*	**19**	**79**	**72.0290**	< 0.0001*	**8**	**34**	**20.3740**	< 0.0001*	**37**	**21**	**6.2170**	**0.0127**	**19**	**8**	**5.1810**	**0.0228**	**79**	**34**	**41.1960**	< 0.0001*

Bold-shadowed cells indicate positive and italics-shadowed cells negative association. Chi-squared tests were used for statistical comparisons. In cases of frequencies lower than 5%, Yates correction was applied. After Bonferroni correction for multiple comparisons, the adjusted alpha level for statistical significance (*) was settled at p < 0.00076.

The trend towards higher pQACV in the right dlPFC-FAT did not reach statistical significance compared to the left side and there were also no statistically significant pQACV differences for ipsilateral comparisons between the SMA-FAT and dlPFC-FAT. An association between increased QA and higher number of total correct responses in the verbal episodic memory task was evidenced involving 15% of the left and 81% of the right SMA-FAT with a significant pQACV lateralization to the right (p < 0.0001, Fig. 3B,C). The pQACV within the dlPFC-FAT was significantly lower in both hemispheres (1% left; 6% right).

**Figure 4:**
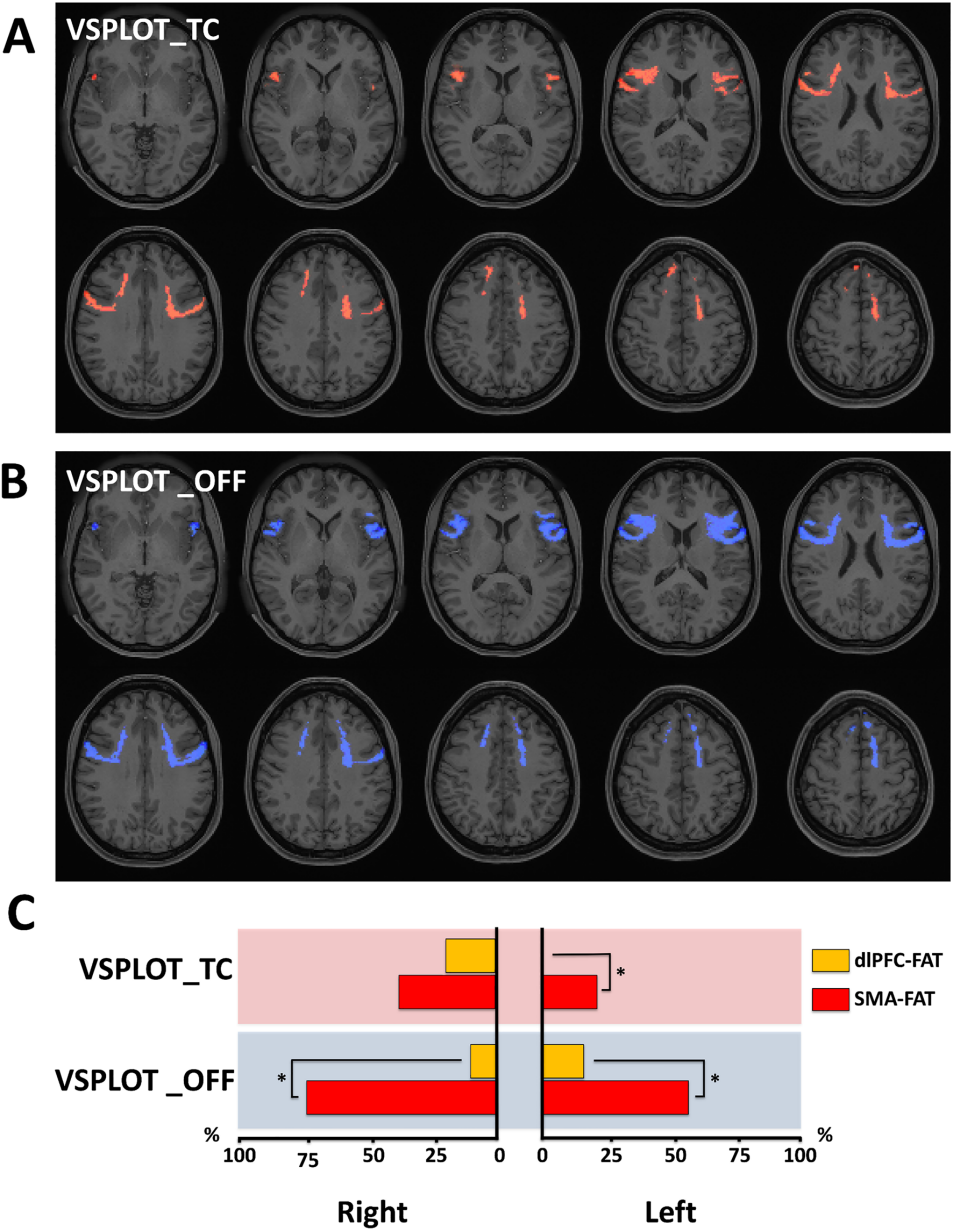
Correlational tractography between FAT-QA-index and the visuospatial orientation test (VSPLOT). Employing diffusion MRI connectometry^56^, a correlation between the QA-index in FAT fibers and visuospatial orientation test scores was derived for the total of 27 subjects. Axial slices in (**A**) and (**B**) illustrate FAT fibers showing association between increased QA and a higher number of correct trials (VSPLOT_TC), and lower total clicks of orientation positions “off” from the correct (VSPLOT_OFF) in the VSPLOT, respectively (red indicating positive and blue indicating negative association). As depicted in (**C**), we calculated the percentual volume of the dlPFC-FAT (orange bars) and SMA-FAT (red bars) showing QA-VSPLOT_TC and QA-VSPLOT_OFF association (pQACV). Pink-shadowed charts indicate positive and blue-shadowed charts negative association. Chi-squared tests were applied for statistical comparisons of pQACV between both FAT subsegments.

Regarding FAT, 31% of the right and 17% of left volumes showed an association between increased QA-index and the number of correct responses in the visual orientation task (Fig. 4A). There was a higher pQACV in the SMA-FAT compared to the dlPFC-FAT, which reached statistical significance for the left hemisphere (p < 0.0001, Fig. 4A,C). In the same way, 52% of the right and 50% of the left FAT evidenced a negative correlation between the QA-index and the total number of orientation positions “off” from the correct one, indicating again an association between higher QA-indexes and better visual orientation performance (Fig. 4B,C).

There was a significantly higher pQACV on the right dlPFC-FAT for total correct responses compared to its homologous on the left side (p < 0.0001). Otherwise, there were no statistically significant differences in pQACV between hemispheres. The pQACV showing association with a better visual orientation performance was significantly higher in the left SMA-FAT compared to the ipsilateral dlPFC-FAT (both p < 0.0001, Fig. 4C). In the right hemisphere, there was only a statistically significant pQACV difference between the SMA-FAT and the dlPFC-FAT in the “off” positions metric (Fig. 4C). The lack of a statistically significant difference between the right SMA-FAT and dlPFC-FAT in total correct responses relied on the notably higher pQACV within the dlPFC-FAT (19%). Regarding visuospatial sustained attention, our analysis failed to show any association between QA-indexes and SPCPT task scores.

**Figure 5:**
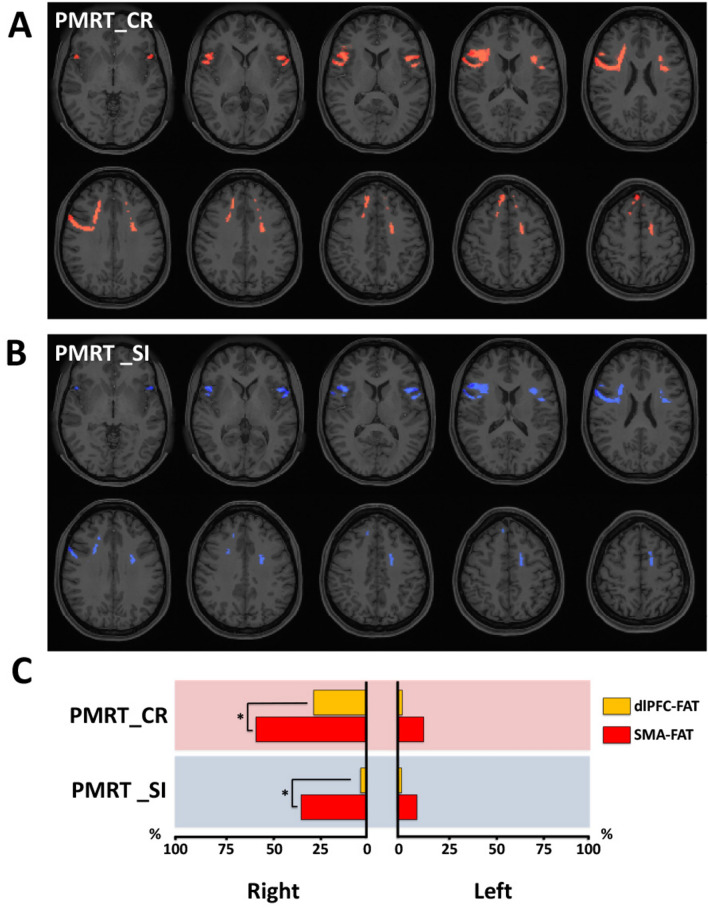
Correlational tractography between FAT-QA-index and fluid intelligence scores. Employing diffusion MRI connectometry^56^, a correlation between the QA-index in FAT fibers and individual fluid intelligence test scores was derived for the total of 27 subjects. Axial slices in (**A**, **B**) illustrate FAT fibers showing association between increased QA and a higher number of correct responses (PMRT_CR), and lower skipped items (PMRT_SI) in the Penn progressive matrices test, respectively (red indicating positive and blue indicating negative association). As depicted in (**C**), we calculated the percentual volume of the dlPFC-FAT (orange bars) and SMA-FAT (red bars) showing QA-PMRT_CR and QA-PMRT_SI association (pQACV). Pink-shadowed charts indicate positive and blue-shadowed charts negative association. Chi-squared tests were applied for statistical comparisons of pQACV between both FAT subsegments.

A higher QA in both FAT, especially in the right hemisphere, was associated with a better fluid intelligence performance (increased number of correct responses and reduced number of skipped items in the PMRT) (Fig. 5). 47% of the right and 12% of the left FAT showed an association between increased QA and a higher number of correct responses in the fluent intelligence task (Fig. 5A,C). The pQACV in the right SMA-FAT was 57% and 27% in the dlPFC-FAT, being both significantly higher than their homologous pQACV on the left hemisphere (13% and 2%, respectively, p < 0.0001). The higher pQACV in the SMA-FAT compared to the ipsilateral dlPFC-FAT reached a statistically significant difference in the right hemisphere (p < 0.0001), remaining the trend below significance on the left side. The association between higher QA and lesser skipped items in the fluent intelligence task was also observed in both hemispheres with right lateralization and SMA-FAT predominance (Fig. 5B,C). Although the overall pQACV on the right (23%) did not reach significance when compared to the left side (9%), a statistically significant difference between sides was observed when comparing the pQACV in the SMA-FAT separately (p < 0.0001). The pQACV within the dlPFC-FAT was scarce (left 2%; right 3%), showing no statistically significant difference between sides.

**Figure 6:**
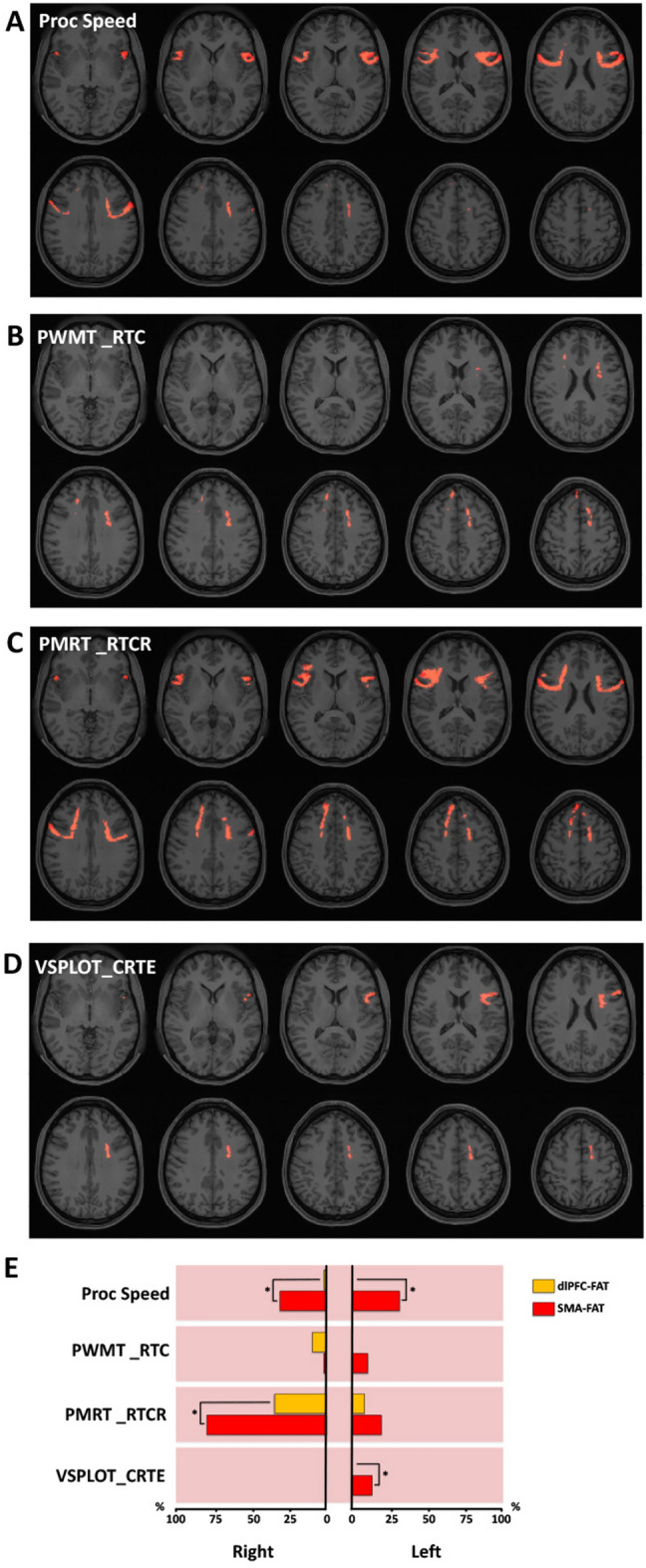
Correlational tractography between FAT-QA-index and processing speed metrics. Employing diffusion MRI connectometry^56^, a correlation between the QA-index in FAT fibers and individual scores in the Age-adjusted NIH toolbox cognitive processing speed test (Proc Speed), as well as in reaction times from the Penn word memory test (PWMT_RTC), Penn progressive matrices reasoning test (PMRT_RTCR) and the Variable short Penn line orientation test (VSPLOT_CRTE) was derived for the total of 27 subjects. Axial slices in (**A**–**D**) illustrate FAT fibers showing association between increased QA and higher scores in Proc Speed, PWMT_RTC, PMRT_RTCR and VSPLOT_CRTE, respectively (red indicating positive association). As depicted in (**E**), we calculated the percentual volume of the dlPFC-FAT (orange bars) and SMA-FAT (red bars) showing an association between QA and the above-mentioned cognitive metrics (pQACV). Pink-shadowed charts indicate positive association. Chi-squared tests were applied for statistical comparisons of pQACV between both FAT subsegments.

There was 26% and 20% pQACV in the left and right FAT, respectively, showing an association between increased QA-index and higher scores in the cognitive processing speed task (Fig. 6A,E). Although no hemispheric lateralization of pQACV was observed, the pQACV was confined almost exclusively to the SMA-FAT. We also found an association between increased QA-index and higher reaction times for correct responses in the verbal episodic memory (Fig. 6B,E), fluid intelligence (Fig. 6C,E) and visuospatial orientation (Fig. 6D,E) tasks. The effect was particularly strong in the fluid intelligence task (involving 17% pQACV in the left and 64% in the right FAT), which was also statistically significant between hemispheres (p < 0.0001). The pQACV in the dlPFC-FAT was proportionally high on the right hemisphere in the verbal episodic memory (9%) and fluid intelligence tests (34%). The pQACV within the SMA-FAT was statistically higher than in the dlPFC-FAT in the right hemisphere in the fluid intelligence test (p < 0.0001), as well as in the left hemisphere in the visuospatial orientation task (p < 0.0006).

## Discussion

The present results indicate a putative relationship, to a different extent, between the microstructural robustness of the FAT and a better performance in verbal and non-verbal episodic memory, spatial orientation, cognitive processing speed and fluid intelligence, but not in visuospatial sustained attention. Furthermore, our data provide evidence that the more posterior portion of the FAT, linked to SMA cortices, plays a more relevant role in the mentioned cognitive functions than the anterior segment innervating areas of the dlPFC. There seems to exist a FAT right hemisphere lateralization in verbal episodic memory processing and fluid intelligence. In contrast, a comparable bilateral pQACV was observed in non-verbal episodic memory, visuospatial orientation and cognitive processing speed. A further novelty in our work was applying QA-based correlational tractography in ultra-high-field 7 T diffusion MRI data to analyze associations between FAT microstructural properties and cognitive performance.

Selecting only highly qualitative ultrahigh-field MRI and cognitive datasets, together with the use of QA-aided correlational tractography, were essential premises in our study seeking to deliver reliable results. QA is based on generalized q-sampling imaging (GQI), which relies upon the Fourier transform relation between diffusion distributions and q-space signals^59^. It enables to filter noisy fibers and defining track terminations, improving deterministic fiber tracking, being less sensitive to partial volume effects of crossing fibers and free water, and serving as an excellent index to define the extent of fiber pathways^59^. Tractography based on QA has been shown to outperform both FA-aided and GFA-aided tractography, providing a better resolution and low false-tracking probability^59,68^. Furthermore, the use of 7 T MRI data has the advantage of outperforming 3 T spatially, increasing the signal-to-noise ratio and improving spatial resolution to detect microstructure changes^69–72^. Also, 7 T diffusion images reduce the uncertainty in fitted values for white matter indexes and the principal eigenvector of the DTI tensor, a fact that makes the usability of white matter anisotropy measures better in fiber tracking applications both in single subject and longitudinal studies^73^. Lastly, the selection of subjects showing full performance in the MMSE and no depressive behavior or emotional lability enabled us to rule out possible confounding biases arising from general cognitive or emotional decline. Following these criteria, we obtained a cohort of well-selected 27 7 T-subjects, which fulfills the nowadays accepted requirements of sample size for such studies, as present evidence demonstrates that a sample of 23 subjects is enough for assessing fraction of anisotropy (a very close microstructural parameter to quantitative anisotropy) within single groups in DTI studies^74^. The subjects' age span was short and varied between 22 and 35 years old. Nevertheless, to rule out possible age-related bias in cognitive performance, only age-adjusted scores were included in the analysis.

Under the above-mentioned methodological conditions, the effect observed in the EM data was consistently present for both tasks and both hemispheres, though stronger for verbal than non-verbal EM. These findings suggest that FAT robustness may constitute a white matter substrate for frontal lobe mechanisms of episodic memory recall^7,75,76^. Although the acquisition of EM has been classically linked to hippocampal activity, the current evidence indicates that the bidirectional connections between medial and ventral regions of the frontal lobe with the thalamus, extrastriate occipital cortex and temporal cortex subserve the access to sensory information to the anterior frontal cortex and a top-down modulation done by the frontal lobe on perceptual processes needed for encoding and retrieval phase of episodic memory tasks and other tasks involving feedback information^25,28,76–78^. There is also evidence showing that the prefrontal cortex retrieves episodic memory from the hippocampus for predicting the outcome of actions based on previous experiences and, in this way, guides the planning of goal-directed behaviors^76^. The match or mismatch of the expected and actual outcomes leads to signals transmitted to the prefrontal cortex, which either consolidates the plan or facilitates a reformulation of the action plan, fostering behavioral flexibility and memory updating. Consistent with this idea, patients with frontal lobe impairments perform poorly on episodic memory tests compared with healthy individuals^33,34^. The data here indicate that the white matter integrity required during the frontal lobe mechanisms of EM recall exerts beyond the solely frontotemporal pathways and purpose that the robustness of more dorsal intralobar frontal connections (i.e., the FAT) may also subserve these mechanisms. This proposal corresponds with previous neuroimaging data on verbal and non-verbal EM, showing activation not only of anterior prefrontal cortices but also in more dorsally located frontal areas corresponding, between others, to the supplementary motor cortices and their connections in the inferior frontal gyrus^29,31,32^. Moreover, our results evidenced that bilateral FAT robustness correlates with both verbal and non-verbal episodic memory performance, and the high percentages of correlation between the right FAT and verbal episodic memory performance are especially interesting. Again, these findings are in line with the previous data on frontal metabolism during EM tasks, which showed bilateral uptake, including a high metabolic activity within the right frontal lobe and prefrontal cortices during verbal EM recall and support the hypothesis that the dorsal portions of the right frontal cortex are involved in monitoring verbal and non-verbal information from the EM in order to make appropriate adaptive responses^29,31,32^.

Additionally, the present results show that bilateral FAT robustness may be relevant in the mechanisms of visual orientation processing but not specifically for visual sustained attention performance. Previous studies have shown the active participation of the FAT in executive visuomotor functions, where visual information subserves goal-directed limb movements and kinematics, such as reaching and grasping^15,16,35,36^. Our results extend these findings suggesting that FAT robustness may be necessary even in earlier stages of planning and execution of visual-driven motor actions, namely during visual processing of the target’s spatial orientation. The results also go in line with previous evidence showing the critical role played by the SMA (the main cortical structure connected by the FAT) in spatial orientation during the Updating task, where the subjects are required to remember their initial orientation while they are passively rotated about their vertical axis in the dark^79^. Together with these previous findings, the data here support the hypothesis of segregated anatomical substrates for two different mechanisms: (1) the manipulation of bodily and visual information for spatial orientation, linked to the FAT and probably subserving its prominent role in the process of planning and executing visually- driven motor actions and (2) the mechanisms for maintaining visual attention on a specific target, primarily relying on frontoparietal connections, such as the superior longitudinal fasciculus^80,81^, but not explicitly related to the FAT. Furthermore, although a trend for higher pQACV in the right FAT was observed in the visual orientation task, a robust bilateral pQACV was present with no clear right-left lateralization. These findings also agree with the bilateral SMA activation on body orientation in the Updating task, as well as with the correlations between white matter robustness indexes in the FAT bilaterally and reaching and reaching-to-grasp scores^16,79^. Hence, although a right dominance is known for visuospatial processing and attention in frontoparietal networks^80,81^, this lateralization seems to become less pronounced in frontal lobe mechanisms of visual information analysis for planning and execution of goal-directed actions.

We observed an important bilateral association between higher QA indexes within the FAT and better performance in the cognitive processing speed task. This observation was consistent throughout the data since a high pQACV was also evidenced in the reaction times for correct responses in EM, fluid intelligence and (though less substantial) in the visuospatial orientation task. Previous studies have emphasized the central role of gray and white matter integrity within both frontal lobes for cognitive processing speed performance over the years^82–84^. Our results complement this evidence proposing the FAT as a relevant white matter substrate associated with cognitive processing speed within the frontal lobe. Given that cognitive processing speed has been shown to facilitate working memory, visuomotor, executive and verbal fluency performance^7,15,37,85–88^, we suggest that FAT involvement in cognitive processing speed may be a main substrate underlying its participation in many other cognitive functions. Altogether, our data postulate the FAT as a primary pathway related to speeding up multimodal information manipulation in the frontal lobes, required for planning and eliciting adequate adaptative behaviors, a fact that agrees with recently evidence showing FAT’s participation in mediating the competition and exclusiveness between internal predictive and external reactive behaviors during sensorimotor tasks^89^. This hypothesis may also partly explain the association found in our data between FAT robustness and better performance in the fluid intelligence test. Fluid intelligence, as the innate ability of an individuum to respond to complex and unexpected situations^90^, has been demonstrated to be strongly influenced by cognitive processing speed and working memory capacity^91–93^. Thus, the role played by the FAT in processing speed, working memory and cognitive control of predictive/reactive behaviors could be partially underlying fluid intelligence performance. This observation agrees with previous works showing that the most robust and most significant associations between white matter robustness indexes and fluid intelligence are in bilateral superior longitudinal fasciculus and bilateral FAT, without significant inter-hemispheric differences^1,90^.

Given that cortical structures associated with the SMA and dlPFC might subserve different (although interrelated) frontal lobe processing networks^53–56^, we segmented the FAT in a more anterior dlPFC-associated component (innervating the 8BL and S6–8 cortices) and a more posterior SMA-associated component (innervating 6ma and SFL cortices). Our results evidenced a significantly larger volume of the SMA-FAT bilaterally, also showing also high percentages of association between increased QA and higher performance in processing speed, visuospatial orientation, EM recall and fluid intelligence tests. These findings support the hypothesis pointing out the role played by the SMA connections with the inferior frontal gyrus on a broader spectrum of cognitive functions beyond language skills and motor planning^94,95^. The somewhat less pronounced white matter component linking the 8BL and S6–8 exclusively with the inferior frontal gyrus may be explained by the fact that dorsolateral prefrontal cortices present an intricated connectional anatomy (involving not only the inferior frontal gyrus but also other prefrontal cortices, the frontal pole, basal ganglia, and temporal, occipital and parietal cortices) related to their participation in highly complex cognitive functions, such as abstract reasoning, critical judgment or metacognition^54,56,77,96–100^. This may also explain the lack of association between QA in specific dlPFC-FAT fibers and the cognitive tasks assessed in this work since the white matter robustness of the dlPFC related to cognitive performance may be linked to a more complex network beyond (and not restricted) to the 8BL/S6–8 connections with the inferior frontal gyrus. Our data also demonstrated also a larger FAT volume (especially the SMA innervating component) in the left hemisphere, consistent with previous findings and attributed to the role played by the FAT and SMA in language and speech^1,9–14^. Interestingly there was no statistical right-left difference in terms of FAT volumes linked to the dlPFC areas, a fact that strengths our proposal that these connections may belong to a more complex not-lateralized dlPFC connectivity network, linked to multimodal and higher order information processing beyond speech and language^54,56,77,96–100^.

The use of 7 T datasets provides the advantage of clearly outperforming 3 T in terms of signal-to-noise-ratio, which impacts the segmentation of the tract and the microstructural properties analysis, but at the cost of having a modest sample size, especially when applying strict inclusion criteria to rule out possible confounding factors. Therefore, future studies are granted, including bigger sample sizes to confirm the data exposed here. However, our results endorse the relevance of FAT robustness in multimodal high-order cognitive performance, a fact with important implications for glioma surgery. For example, in young and middle-aged patients with IDH-mutant gliomas, the current initial treatment, including gross total resection followed by radiation and chemotherapy, may achieve longer progression-free and overall survival rates^101,102^. Therefore, preservation of cognition is fundamental for enabling the patients to reassume their daily social and professional activities, all relevant aspects for their long-term quality of life. Thus, the current study paves the way for further research required to determine to which extent the damage of the FAT and its subsegments could lead to potentially harmful cognitive sequela, its recovery potential, and to which extent supramarginal resections of tumors located next to FAT should be pushed, considering tumor characteristics, prognostic factors and patient expectations.

## Conclusions

The results here presented go beyond the already existing evidence and demonstrate that both the right and left FAT constitute a main connecting pathway within the frontal lobe, subserving many other cognitive neocortical domains besides speech fluency, working memory or visuomotor executive functions. Our data suggest that FAT microstructural robustness, especially its SMA-linked component, may also be playing a relevant role, to a different extent, in EM recall, visuospatial orientation and cognitive processing speed, even underlying individual fluid intelligence. In light of the present evidence, further prospective research should be encouraged to better characterize a possible impact caused by FAT damage on these cognitive functions (as well as their potential for recovery) in order to provide more straightforward recommendations for neurosurgeons dealing with lesions next to this white matter bundle, especially during supramarginal resections of prefrontal gliomas.

### Supplementary Information

Supplementary Information.

## Data Availability

The data that support the findings of this study are available from the Human Connectome Project, WU-Minn Consortium (principal investigators: D. Van Essen and K. Ugurbil; 1U54MH091657) funded by the 16 NIH institutes and centers that support the NIH Blueprint for Neuroscience Research; and by the McDonnell Center for Systems Neuroscience at Washington University but restrictions apply to the availability of these data (users must agree to data use terms for the HCP before being allowed access to the data). Data are however available from the authors upon reasonable request and with permission of the Human Connectome Project.
