# Supplementary material for: Harnessing the frontal aslant tract’s structure to assess its involvement in cognitive functions: new insights from 7-T diffusion imaging
Source: Sci Rep. 2024 Jul 29;14:17455. doi: 10.1038/s41598-024-67013-w (PMC11286763; doi:10.1038/s41598-024-67013-w)
Supplement: Supplementary file 1 — Supplementary Information. [file 41598_2024_67013_MOESM1_ESM.docx]

**Supplementary information (Serrano Sponton et. al.)**

Scores descriptions are taken from https://db.humanconnectome.org/

**Mini Mental Status Examination (MMSE) Total Score^1^**

Mini Mental Status Examination (MMSE) Total Score. This is a broad measure of cognitive status. The maximum score for the MMSE is 30, being scores of 25 or higher considered as normal. Scores above 28 indicate intact cognition. If the score is below 24, the result is usually considered to be abnormal, indicating possible cognitive impairment.

**NIH Toolbox Sadness Survey^2^**

This self-report measure assesses sadness for ages 18-85 using a CAT format. The Unadjusted Scale Score has a Mean of 50, SD of 10. Higher scores are indicative of more sadness. Scores 1 SD or more below the mean (T ² 40) suggest low levels of sadness and scores 1 SD or more above the mean (T ³ 60) suggest high levels of sadness.

## NIH Toolbox General Life Satisfaction Survey^2^

This self-report measure assesses global feelings and attitudes about one's life using a CAT format. The Unadjusted Scale Score has a Mean of 50, SD of 10. Higher scores are indicative of more general life satisfaction. Scores 1 SD or more below the mean (T ² 40) suggest low levels of general life satisfaction and scores 1 SD or more above the mean (T ³ 60) suggest high levels of general life satisfaction.

## NIH Toolbox Meaning and Purpose Survey^2^

This self-report measure for adults (ages 18 and above) is a CAT that accesses the extent to which people feel their life matters or makes sense. The Unadjusted Scale Score has a Mean of 50, SD of 10. Higher scores indicate more self-reported meaning and purpose. Scores 1 SD or more below the mean (T ² 40) suggest low levels of meaning and purpose and scores 1 SD or more above the mean (T ³ 60) suggest high levels of meaning and purpose.

## NIH Toolbox Positive Affect Survey^2^

This self-report measure assesses both activated (i.e., happiness, joy) as well as unactivated (i.e., serenity, peace) aspects of positive affect in CAT form for ages 18-85. The Unadjusted Scale Score has a Mean of 50, SD of 10. Higher scores are indicative of more positive affect. Scores 1 SD or more below the mean (T ² 40) suggest low levels of positive affect and scores 1 SD or more above the mean (T ³ 60) suggest high levels of positive affect.

**NIH Toolbox Loneliness Survey^2^**

This self-report measure assesses perceptions of loneliness using a 5-item fixed-length form for ages 18-85. The Unadjusted Scale Score has a Mean of 50, SD of 10. Higher scores are indicative of more loneliness. Scores 1 SD or more below the mean (T ² 40) suggest low levels of loneliness and scores 1 SD or more above the mean (T ³ 60) suggest high levels of loneliness.

**Refereces:**

1. Folstein MF, Folstein SE, McHugh PR. "Mini-mental state". A practical method for grading the cognitive state of patients for the clinician. J Psychiatr Res. 1975 Nov;12(3):189-98.
2. NIH Toolbox for assessment of neurological and behavioral function. Neurology 2013 Mar12;80 (11 Supplement 3)
